# P02.80. Safety and feasibility of modified chair-yoga on functional outcome among seniors at risk for falls

**DOI:** 10.1186/1472-6882-12-S1-P136

**Published:** 2012-06-12

**Authors:** M Galantino, L Greene, R Marsico, K Desai, J DeCesari, N MacKain, S Rinaldi, M Stevens, V Wurst, J Mao

**Affiliations:** 1 The Richard Stockton College of New Jersey, Galloway, USA; 2 University of Pennsylvania, Philadelphia, USA

## Purpose

Falls are among the most common problems affecting older adults. At least 50% of those over the age of 80 fall annually. The goal of this pilot was to assess the safety and feasibility of structured yoga in an elderly population with fall risk.

## Methods

Seniors at risk for falls were identified and enrolled in a single arm pilot trial. A chair based yoga program was provided twice a week for 8 weeks. The program was designed from previous published pilot data. A battery of validated instruments was administered at baseline and week eight and was used to identify which instrument may be sensitive to change as a result of a yoga program.

## Results

Among sixteen seniors (median age of 88) with a previous history of falls, 87% provided data for assessment at the end of the intervention. Two patients withdrew, one due to a fall outside the institution and the other due to lack of time and interest. There were no adverse events during the yoga sessions. Paired-t tests compared pre-post changes and gains were noted in Fear of Falling (5.27 to 2.60; p=0.029) and the SPPB sit to stand subscale (0.31 to 1.00; p=.022). Improved trends were noted in the anxiety subscale of the Hospital Anxiety and Depression (6.10 to 4.86, p=.072) and the Timed Up and Go (22.57 to 18.97, p= 0.052) assessments.

## Conclusion

We found the modified chair-yoga program is safe and recruitment is feasible. To provide a skillful framework for teaching yoga to seniors, specific principles of practice are important to ensure safety of this intervention. Our data suggest that yoga may be beneficial in improving mobility and reducing fear of falling which warrants additional research via randomized controlled trials.

